# An obstacle to overcome: cerebral toxoplasmosis in patients living with HIV

**DOI:** 10.1186/1471-2334-14-S4-P48

**Published:** 2014-05-29

**Authors:** Irina Filipescu, Roxana Iubu, Cristian Marcu, Violeta Năstase, Mirela Flonta, Ligia Ursu, Corina Itu

**Affiliations:** 1 Iuliu Hațieganu University of Medicine and Pharmacy, Cluj-Napoca, Romania; 2 Infectious Diseases Hospital, Cluj Napoca, Romania

## 

Toxoplasmosis represents the most frequent complication affecting the CNS of AIDS patients.

In Europe and South America the prevalence of toxoplasmosis is higher than in the United States (50-75% to 15%), so the risk for the AIDS-associated toxoplasmosis is higher in this area, including Romania.

Most of the AIDS patients have immunoglobulin G antibodies anti-*Toxoplasma* in their serum, like in the general population, so most of the cases represent a reactivation of a latent infection.

We present a series of 5 cases with patients, aged between 19-29 years old, who developed cerebral toxoplasmosis.

Three of the patients are multi-experienced, and for two patients the cerebral toxoplasmosis was the defining AIDS infection.

Three patients had a CD4 count under 100 cells/cmm at the moment of their diagnosis, with low adherence for cART and for the prophylaxis with trimethoprim-sulfamethoxazole.

All patients but one had detectable HIV viral load.

Two of the patients are positive for B hepatitis, one of them also for hepatitis D.

The clinical manifestations were persistent headache, confusion, lethargy, hemiparesis.

All patients presented high levels for immunoglobulin G anti-*Toxoplasma* at the moment of the clinical manifestations, one patient presented immunoglobulin M anti-*Toxoplasma*.

The MRI examinations revealed characteristic multiple lesions.

The treatment was performed using trimethoprim-sulfamethoxazole for 6 weeks.

The evolution of the patients under the treatment was favorable for all the patients, with the remission of the symptoms and without neurological complications; they received trimethoprim-sulfamethoxazole prophylaxis until their CD4 >200/cmm, for another three months

Although difficult to diagnose, cerebral toxoplasmosis is treatable and curable, despite of all the associated AIDS pathology.

